# Whole genome duplication of wild-type and *CINNAMYL ALCOHOL DEHYDROGENASE1*-downregulated hybrid poplar reduces biomass yield and causes a brittle apex phenotype in field-grown wild types

**DOI:** 10.3389/fpls.2022.995402

**Published:** 2022-09-09

**Authors:** Marlies Wouters, Sander Corneillie, Angelo Dewitte, Jan Van Doorsselaere, Jan Van den Bulcke, Joris Van Acker, Bartel Vanholme, Wout Boerjan

**Affiliations:** ^1^Department of Plant Biotechnology and Bioinformatics, Ghent University, Ghent, Belgium; ^2^VIB Center for Plant Systems Biology, Ghent, Belgium; ^3^Expertisecentrum Agro- en Biotechnologie, VIVES, Roeselare, Belgium; ^4^Laboratory of Wood Technology, Department of Environment, Faculty of Bioscience Engineering, Ghent University, Ghent, Belgium

**Keywords:** polyploidy, field trial, CAD, lignin, saccharification

## Abstract

The potential of whole genome duplication to increase plant biomass yield is well-known. In Arabidopsis tetraploids, an increase in biomass yield was accompanied by a reduction in lignin content and, as a result, a higher saccharification efficiency was achieved compared with diploid controls. Here, we evaluated whether the results obtained in Arabidopsis could be translated into poplar and whether the enhanced saccharification yield upon alkaline pretreatment of hairpin-downregulated *CINNAMYL ALCOHOL DEHYDROGENASE1* (*hpCAD*) transgenic poplar could be further improved upon a whole genome duplication. Using a colchicine treatment, wild-type (WT) *Populus tremula* x *P. alba* cv. INRA 717-1B4, a commonly used model clone in tree biotechnology research, and *hpCAD* tetraploids were generated and grown in the greenhouse. In parallel, WT tetraploid poplars were grown in the field. In contrast to Arabidopsis, a whole genome duplication of poplar had a negative impact on the biomass yield of both greenhouse- and field-grown trees. Strikingly, field-grown WT tetraploids developed a brittle apex phenotype, i.e., their tip broke off just below the apex. In addition, the chromosome doubling altered the biomass composition of field-grown, but not of greenhouse-grown tetraploid poplars. More specifically, the lignin content of field-grown tetraploid poplars was increased at the expense of matrix polysaccharides. This increase in lignin deposition in biomass is likely the cause of the observed brittle apex phenotype, though no major differences in stem anatomy or in mechanical properties could be found between di- and tetraploid WT poplars grown in the field. Finally, without biomass pretreatment, the saccharification efficiency of greenhouse- and field-grown WT diploids was not different from that of tetraploids, whereas that of greenhouse-grown *hpCAD* tetraploids was higher than that of greenhouse-grown diploids. Upon alkaline pretreatment, the saccharification yield of diploids was similar to that of tetraploids for all genotypes and growth conditions tested. This study showed that a whole genome duplication in hybrid WT and *hpCAD* poplar did neither result in further improvements in biomass yield, nor in improved biomass composition and, hence, saccharification performance.

## Introduction

Poplar (*Populus* spp.) is an ecologically and economically important tree genus widely grown in the northern hemisphere. Because of their fast growth, easy clonal propagation and amenability to genetic transformation, poplars have become the species of choice for genetics, physiology and biotechnology research on trees (Wullschleger et al., 2002; Singh et al., 2021). In addition, poplar woody biomass is increasingly gaining interest as a renewable feedstock for biorefineries, explaining the research efforts focusing on its secondary cell wall (Porth, 2015).

The plant secondary cell wall consists of large amounts of polysaccharides embedded in lignin. These polysaccharides can be enzymatically depolymerized—a process called saccharification—into monosaccharides that, in turn, can be fermented into yield biofuels or building blocks for all kinds of biobased products that are nowadays predominantly made from fossil sources (Vanholme et al., 2013). The major factor limiting efficient saccharification is lignin, hindering the access of depolymerizing enzymes to the polysaccharides (Chen and Dixon, 2007; Van Acker et al., 2013; Zeng et al., 2014). Lignin is a heterogeneous aromatic polymer composed of H, G and S units that are formed by combinatorial radical coupling of the monolignol building blocks *p*-coumaryl, coniferyl and sinapyl alcohol, respectively (Boerjan et al., 2003; Bonawitz and Chapple, 2010; Vanholme et al., 2019). Reducing the lignin amount or altering its composition by genetic engineering is a popular strategy to improve the saccharification efficiency of the lignocellulosic biomass (Chanoca et al., 2019; Bryant et al., 2020). However, reducing lignin content has its limit as this polymer plays a role in providing fiber strength and vessel hydrophobicity to the cell wall, ultimately resulting in a drop in biomass yield when lignin levels are too low (Muro-Villanueva et al., 2019; De Meester et al., 2020; De Vries et al., 2021). To fully unlock the potential of trees as biorefinery crops, both biomass yield and cell wall composition should be optimized. Using traditional breeding, such as interspecies hybridization, large improvements in biomass yield have been obtained, and genetic engineering strategies have proven to be successful in lowering the lignin amount without yield penalty (Van Acker et al., 2017; De Meester et al., 2020, 2021; Gui et al., 2020; Jang et al., 2021). The induction of artificial polyploidization might be an additional option to further improve the biomass yield and saccharification efficiency of trees.

Polyploidy typically increases cell size, known as the “*gigas”* effect of polyploidy (Stebbins, 1971). *In vitro* induction of allotetraploids in *P. alba x P. glandulosa* and (*P. pseudo-simonii x P. nigra*) *x P. beijingensis* resulted in larger and thicker leaves (Xu et al., 2016; Ren et al., 2021). In *Arabidopsis thaliana*, it has been found that artificial autotetraploids have a significant increase in stem height and dry weight, while the lignin content is reduced, resulting in an improved saccharification efficiency (Corneillie et al., 2019). A similar correlation between ploidy level and lignin content has been described for autotetraploid shrub willow, although the saccharification potential of the obtained biomass was not determined (Serapiglia et al., 2014, 2015). It has also been reported for autotetraploid acacia that the Kraft pulp yield is similar to that of diploid wood, but less alkali is consumed, indicating improved wood processing efficiency (Griffin et al., 2014).

To investigate the potential of polyploidization as a means to improve both the biomass yield and saccharification efficiency of poplar, colchicine-mediated induction of tetraploids was performed in the wild-type (WT) female hybrid *P. tremula* x *P. alba* cv. INRA 717-1B4, a well-studied model clone because of its high amenability to *Agrobacterium*-mediated transformation and the availability of a genome sequence (Leplé et al., 1992; Chupeau et al., 1994; Busov et al., 2011; Mader et al., 2016; Nietsch et al., 2017), and the transgenic hairpin-downregulated *CINNAMYL ALCOHOL DEHYDROGENASE1* (*hpCAD*), made in the same genetic background (Van Acker et al., 2017). CAD1 catalyzes the last step of the monolignol biosynthesis pathway, and downregulating *CAD1* in *P. tremula* x *P. alba* reduces the conversion of hydroxycinnamaldehydes to their respective alcohols. Consequently, sinapaldehyde is incorporated in the lignin of *CAD1*-downregulated poplars resulting in (1) a higher proportion of free phenolic end-groups, making lignin more alkali-soluble, and (2) an increase in conjugated carbonyl functions that facilitate lignin cleavage under alkaline conditions (Baucher et al., 1996; Pilate et al., 2002; Lapierre et al., 2004; Van Acker et al., 2017). Accordingly, the saccharification yield of greenhouse-grown *hpCAD* poplars is increased by up to 81% compared with non-transgenic control lines upon an alkaline pretreatment (Van Acker et al., 2017).

Here, we studied the effect of whole genome duplication on the biomass yield, biomass composition and saccharification efficiency of WT and *hpCAD* allotetraploids that were grown in greenhouse conditions. In addition, a field trial was performed with the WT allotetraploids and their diploids controls.

## Materials and methods

### Plant material

The lines used were WT hybrid *P. tremula* x *P. alba* cv. INRA 717-1B4 and transgenic *hpCAD* line 19 as described in Van Acker et al. (2017).

### Induction of polyploidy

Tetraploids were generated using a colchicine treatment. In brief, leaves of *in vitro* plantlets were removed and stems were cut in 1-cm pieces, each containing an axillary bud. Next, stem pieces were incubated (while shaking) in a 0.1% colchicine solution for 24 h, after which they were rinsed with sterile H_2_O and transferred to M1/2 propagation medium [2.2 g/L MS; 1 mg/L cysteine; 0.2 g/L glutamine; 20 g/L sucrose; 5.5 g/L agar; 1% (v/v) Morel & Wetmore vitamins; 0.5 mg/L IAA; pH 5.9; adapted from Leplé et al. (1992)], allowing each axillary bud to grow into an independent *in vitro* plantlet.

### Determination of the ploidy level

From the pool of colchicine-treated plantlets, diploid lines (2n = 2x), of which the treatment was not effective, and tetraploid lines (2n = 4x), of which the treatment was effective, were selected. The somatic ploidy level of all plantlets was determined using DNA flow cytometry, as described in Corneillie et al. (2019), and confirmed upon individual chromosome counting of chromosome spreads. Chromosome spreads were prepared according to Kirov et al. (2014) with an enzyme treatment of 1% cellulase and 1% pectolyase for 75 min.

### Growth of trees in the greenhouse and the field, and biomass yield analyses

Three independent tetraploid WT lines and *hpCAD* lines—each with their diploid control line—were selected for analysis. Each line was *in vitro* propagated in clonal replicates, i.e., stems were cut into 1-cm stem pieces containing an axillary bud and each piece put on M1/2 medium (Leplé et al., 1992). After rooting, plantlets were transferred to soil. In the greenhouse (GH), 15 clonal replicates per line (di- and tetraploid WT and *hpCAD* lines) were grown in a randomized design. One *hpCAD*_4x biological replicate died and was left out the analyses. After an initial growth of 2 months in soil, all plants were cut back to ensure equal height of all trees at the start of the measurements. After resprouting from the stool, all shoots but the main new one were removed. The height of the main stem was monitored on a weekly basis for a period of 120 days. The growth speed was calculated by dividing the difference between the final height at day 120 and the initial height at day 21 by the analyzed period (99 days). At harvest time (day 120), the final height, the stem diameter 50 cm above soil level and the total dry weight of the debarked tree were measured.

In the field (F), 18 clonal replicates per di- and tetraploid WT line were grown in a randomized block design consisting of three blocks, each containing six clonal replicates per line (Supplementary Figure 1). The poplars were grown for 3 months in the greenhouse before they were planted at the field site located in Zwijnaarde, Belgium (51°00′00″ N, 3°42′60″ E) in May 2016. One WT_4x biological replicate snapped during planting and was left out the analyses. The height of the main stem was monitored at 11 time points over a period of 170 days. All trees that showed the brittle apex phenotype were excluded from further analyses of biomass yield. The growth speed was calculated by dividing the difference between the height at day 170 and day 9 by the period being analyzed (161 days). At day 170, the final height was measured. At harvest time in January 2017, after one growth season, the stem diameter 50 cm above soil level and the total dry weight of the debarked tree were measured.

### Biomass composition analyses

All cell wall analyses were performed on purified cell wall residue (CWR) of the debarked bottom 50-cm part of the main stem harvested 10 cm above soil level. A subset of trees was made of which the dry stem weight was the closest to the average dry weight of that line (eight trees per line) for greenhouse-grown trees harvested at day 120; nine trees per line (three trees per block) for field-grown trees harvested in January 2017. A purified CWR of ground and sieved (0.5-mm mesh) stem material was prepared using sequential extraction steps as described in Van Acker et al. (2013). The crystalline cellulose content was determined based on Updegraff (1969) as reported by Foster et al. (2010). Matrix polysaccharides (MPS, i.e., hemicellulose and pectin) were extracted from the CWR with 2 M trifluoroacetic acid (TFA) for 2 h at 99°C while shaking (750 rpm), dried under vacuum and weighed. The different monosaccharides present in the TFA extract were quantified *via* gas chromatography-mass spectrometry (GC-MS) as their corresponding alditol acetates as described by Foster et al. (2010). Response factors for each of the monosaccharides were taken from Van Acker et al. (2013). The lignin content was measured gravimetrically *via* the Klason method as essentially described by Ibáñez and Bauer (2014) and modified by Saleme et al. (2017). Klason data of greenhouse- and field-grown poplars were collected in different experiments and, therefore, cannot be compared. The lignin composition (traditional lignin units and S aldehyde marker for CAD1 deficiency) were quantified using thioacidolysis *via* GC-MS as their trimethylsilyl ether derivatives according to Robinson and Mansfield (2009). Response factors for H, G and S units, and the S aldehyde marker for CAD1 deficiency were taken from Van Acker et al. (2013). Thioacidolysis data of WT and *hpCAD* greenhouse-grown and WT field-grown poplars were collected in different experiments and, hence, cannot be compared.

### Biomass saccharification assays

Saccharification assays were performed on the debarked bottom 50-cm part of the main stem of a subset of trees (see above) according to Van Acker et al. (2016). For alkaline pretreatment, ground and sieved (0.5-mm mesh) stem material was incubated in 62.5 mM NaOH for 3 h at 90°C. The enzyme mix added to each sample contained cellulase (from *Trichoderma reesei* ATCC 26921, Sigma Aldrich) and beta-glucosidase (Accellerase BG, Novozyme). Both enzymes were desalted over an Econo-Pac 10 DG column (Bio-Rad) and stacked with Bio-gel^®^ P-6 DG gel (Bio-Rad) according to the manufacturer's guidelines. The activity of the enzyme mix was determined with a filter paper assay as described by Xiao et al. (2004). An activity of 0.01 filter paper units was added to each sample. Released glucose was measured after 10 h of saccharification *via* a quantitative color reaction and expressed as a percentage of dry weight. Saccharification data of greenhouse- and field-grown poplars were collected in different experiments and, hence, cannot be compared.

### Microscopy

For microscopy, the main stem of field-grown WT trees was harvested 1 m above soil level in August 2017 of the second growth season. At that time, the WT tetraploids already showed the brittle apex phenotype. Harvested parts were fixed in 70% (v/v) ethanol. Multiple transversal stem sections of 20 μm thickness were made per plant using a Reichert-Jung 2040 Autocut Microtome (Leica) and stained with 0.5% (w/v) astra blue, 0.5% (w/v) chrysiodine and 0.5% (w/v) acridine red for 10 min. To prepare the triple staining solution, 0.5% (w/v) astra blue (Santa Cruz Biotechnology) in 2% tartaric acid was mixed with 0.5% (w/v) chrysoidine (Sigma Aldrich) in 5% (w/v) ammonium aluminum sulfate and 0.5% (v/v) glacial acetic acid and 0.5% (w/v) acridine red (Santa Cruz Biotechnology) in 5% ammonium aluminum sulfate and 0.5% (v/v) glacial acetic acid in a 4:1:1 ratio. After dehydration in isopropyl alcohol, the stained sections were mounted in Euparal mounting medium (Carl Roth). Images were acquired using a Zeiss Axioskop 2 microscope and processed using automated software described by Andriankaja et al. (2012) to quantify the average number and average area of vessels and fibers per selected area. The number of vessels was divided by the number of fiber cells to provide a ratio. The proportion of vessel lumen was defined as the total vessel area per selected area.

### Three-point bending test

For the tree-point bending test, samples of the main stem of field-grown WT trees of approximately 50-cm in length were harvested in February 2022, when the trees were 4 years old. We sampled at the top and at the base of the stem, respectively, of di- and tetraploid poplars to obtain an as comparable as possible stem diameter range across all samples. The stem samples were debarked and dried at 40°C for 4 weeks. Three-point bending tests were performed using a universal testing machine (ZwickRoell). The span between the supporting pins was 340 mm, a pre-load of 30 N was applied and the actual bending was performed with a constant displacement of 8 mm/min of the central indenting pin until sample failure (30% decrease of the maximum applied force). The force-displacement data were exported to Excel.

The modulus of elasticity (MOE) was calculated using **Equation 1**:


$$\begin{array}{l} MOE\;=\;\frac{sL^{3}}{12\pi r^{4}}\;\left(MPa\right) \end{array}$$

with s the slope of the force-displacement curve in the elastic region (100 N–400 N), L the span between the supporting pins and r the radius of the stem sample (an average from two measurements taken in the center of the sample where the force by the central indenting pin was applied).

The modulus of rupture (MOR) was calculated using **Equation 2**:


$$\begin{array}{l} MOR\;=\;\frac{8FL}{\pi d^{3}}\;\left(MPa\right) \end{array}$$

with F the maximum applied force at failure, L the span between the supporting pins and d the diameter of the sample (a measurement taken in the center of the sample where the force by the central indenting pin was applied).

### Statistical analyses

Greenhouse and field data were statistically analyzed by fitting (generalized) linear (mixed) models to the data. For a more detailed description of the models and the statistical tests, see Supplementary Materials and Methods 1.

## Results

### Generation of allotetraploid WT and *hpCAD* poplar lines

**Figure 1:**
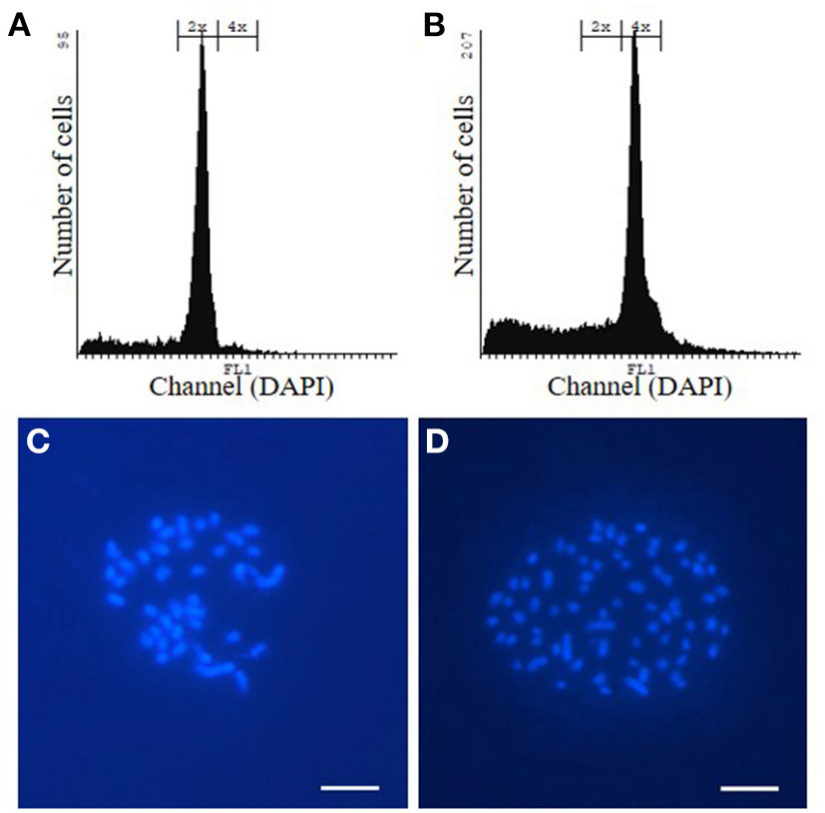
Confirmation of the ploidy level of WT poplar plantlets grown from colchicine-treated axillary buds *via* flow cytometry and chromosome spreads. **(A, C)** WT diploid poplar. **(B, D)** WT tetraploid poplar. Pictures are representative for the different independent tetraploid lines. The same way, the ploidy level of *hpCAD* di- and tetraploid poplar was verified (Supplementary Figure 2). Scale bars = 5 μm.

Allotetraploid *P. tremula x P. alba* WT and *hpCAD* poplar were obtained by incubating *in vitro* axillary buds of diploid plantlets with colchicine, an anti-mitotic agent. Subsequently, plantlets were grown from the colchicine-treated axillary buds and their ploidy levels were determined *via* flow cytometry (Figures 1A,B; Supplementary Figure 2). For both the WT and *hpCAD* genetic background, three tetraploid plantlets were clonally propagated as independent lines. All three tetraploid WT and *hpCAD* lines were grown in the greenhouse alongside their diploid control lines in a randomized design (15 trees per line). Simultaneously, a field trial was initiated with di- and tetraploid WT poplar lines (18 trees per line). Because transgenic *hpCAD* poplars need a regulatory permit to be tested in the field, we limited the field trial to the tetraploid WT lines and the corresponding diploid controls. The trees were planted in the field in May 2016 according to a randomized block design (three blocks of six trees per line; Supplementary Figure 1). In the meantime, to confirm the ploidy level of the selected lines, chromosome spreads were made to count the number of chromosomes (Figures 1C,D; Supplementary Figure 2). In line with the wild-type haploid chromosome number of 19 (Blackburn and Harrison, 1924), the chromosome numbers of di- and tetraploid lines were 38 and 76, respectively. However, one clonally propagated WT line turned out to be composed of both di- and tetraploid cells. Therefore, this mixoploid line—although initially planted in the greenhouse and field—was excluded from further analysis.

### Biomass yield is reduced in greenhouse and field-grown allotetraploid poplars

**Figure 2:**
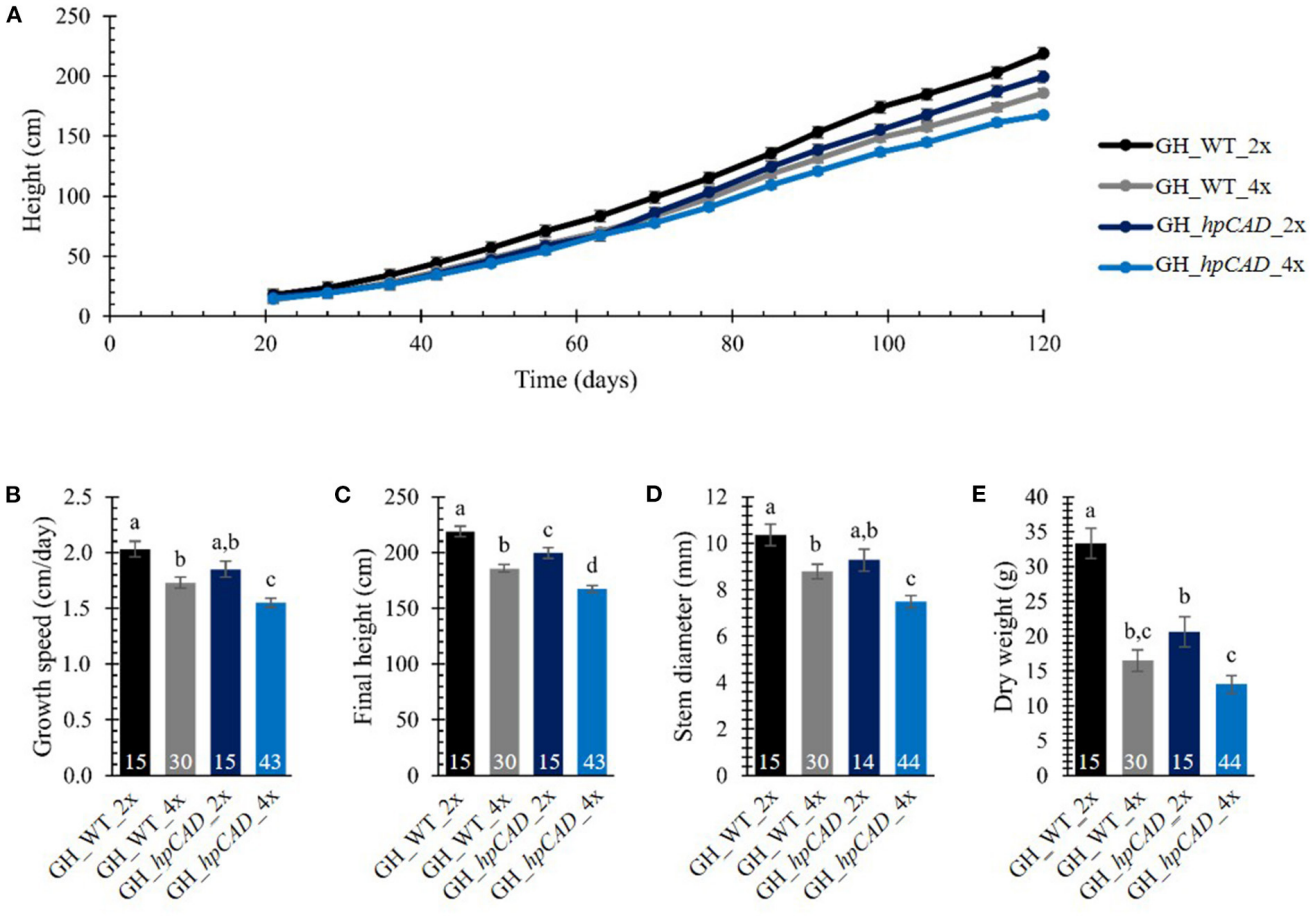
Biomass yield of greenhouse-grown di- and tetraploid poplar trees. **(A)** Height over time. The height of the main stem was measured on a weekly basis over a period of 120 days. **(B)** Growth speed. **(C)** Final height of the poplars measured at day 120. **(D)** Stem diameter measured at harvest time 50 cm above soil. **(E)** Total dry weight of the debarked tree measured at harvest time. The number of biological replicates is indicated in the bars. Data shown are adjusted mean ± standard error of the mean (SEM). Different letters represent significant differences at the 0.05 significance level. The significance differences for (A) are provided in Supplementary Table 1. For a detailed description of the models fitted to the data and the statistical tests used, see Supplementary Materials and Methods 1.

To evaluate the effect of polyploidization on the biomass yield of hybrid *P. tremula x P. alba* trees, the greenhouse-grown trees were cut back after 2 months of growth to ensure uniform regrowth for the subsequent growth measurements. The height of the main stem resprouting from the stool was measured on a weekly basis over a period of 120 days (Figure 2A; Supplementary Table 1). As growth characteristics of the tetraploid lines with the same background (i.e., WT or *hpCAD*) were indistinguishable from one another over the period of observation, they were combined for data presentation and termed GH_WT_4x (30 replicates) and GH_*hpCAD*_4x (44 replicates).

First, we addressed the effect of whole genome duplication on biomass yield parameters in greenhouse-grown WT and *CAD1*-downregulated poplars. For WT trees, a decrease in height of tetraploids (vs. diploids) was apparent from day 63 onwards, whereas for *hpCAD* tetraploid trees, the difference was only significant from day 77 onwards. The decrease in height upon polyploidization was attributed to a reduction in growth speed of 14.78% for WT tetraploids and 16.22% for *hpCAD* tetraploids (Figure 2B; Supplementary Table 2). Accordingly, at the time of harvest (day 120), a negative impact of polyploidization on the final height was observed in the WT background (reduction of 15.11%) and the *hpCAD* background (reduction of 16.01%, Figure 2C; Supplementary Table 2). In contrast to their diploid counterparts, the stem diameter measured 50 cm above soil was 15.22% smaller in WT tetraploids and 19.48% smaller in *hpCAD* tetraploids at harvest time (Figure 2D; Supplementary Table 2). In addition, the total dry weight of the debarked trees at harvest time was reduced by 50.54% and 36.53% in WT and *hpCAD* tetraploids, respectively, compared with their diploid counterparts (Figure 2E; Supplementary Table 2).

**Figure 3:**
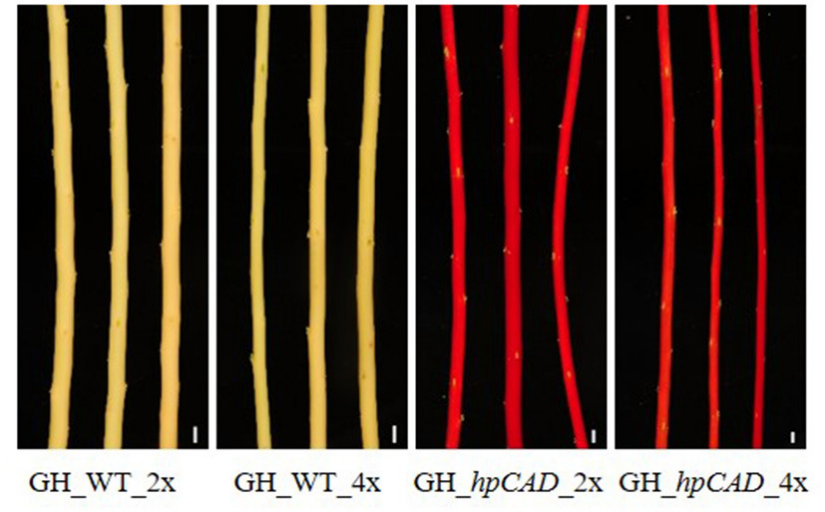
Xylem coloration of greenhouse-grown di- and tetraploid debarked poplar stems. The red xylem phenotype characteristic for *CAD1*-downregulation is maintained after whole genome duplication. Pictures are representative for the different independent lines. Scale bars = 1 cm.

Next, we studied the consequence of *CAD1*-downregulation. As a result of *CAD1*-downregulation, both *hpCAD* di- and tetraploid poplars started to show growth retardation from day 91 onwards, resulting in a reduction of the final height of respectively 8.85% and 9.82% compared with their WT counterparts (Figures 2A,C; Supplementary Tables 1, 2). Although the other parameters of biomass yield showed a similar trend for *CAD1*-downregulated trees, only the growth speed (reduction of 10.44%) and the diameter (reduction of 14.87%) of *hpCAD* tetraploids were significantly decreased compared with those of the WT tetraploids (Figures 2B,D; Supplementary Table 2). Inversely, only the total dry weight of the debarked trees (reduction of 38.14%) of *hpCAD* diploids was significantly lower compared with WT diploids (Figure 2E; Supplementary Table 2). Furthermore, debarked stems of the diploid *hpCAD* trees had the reddish colored xylem characteristic for *CAD1*-downregulated trees (Baucher et al., 1996; Van Acker et al., 2017), and this phenotype was maintained in the corresponding tetraploid lines (Figure 3).

**Figure 4:**
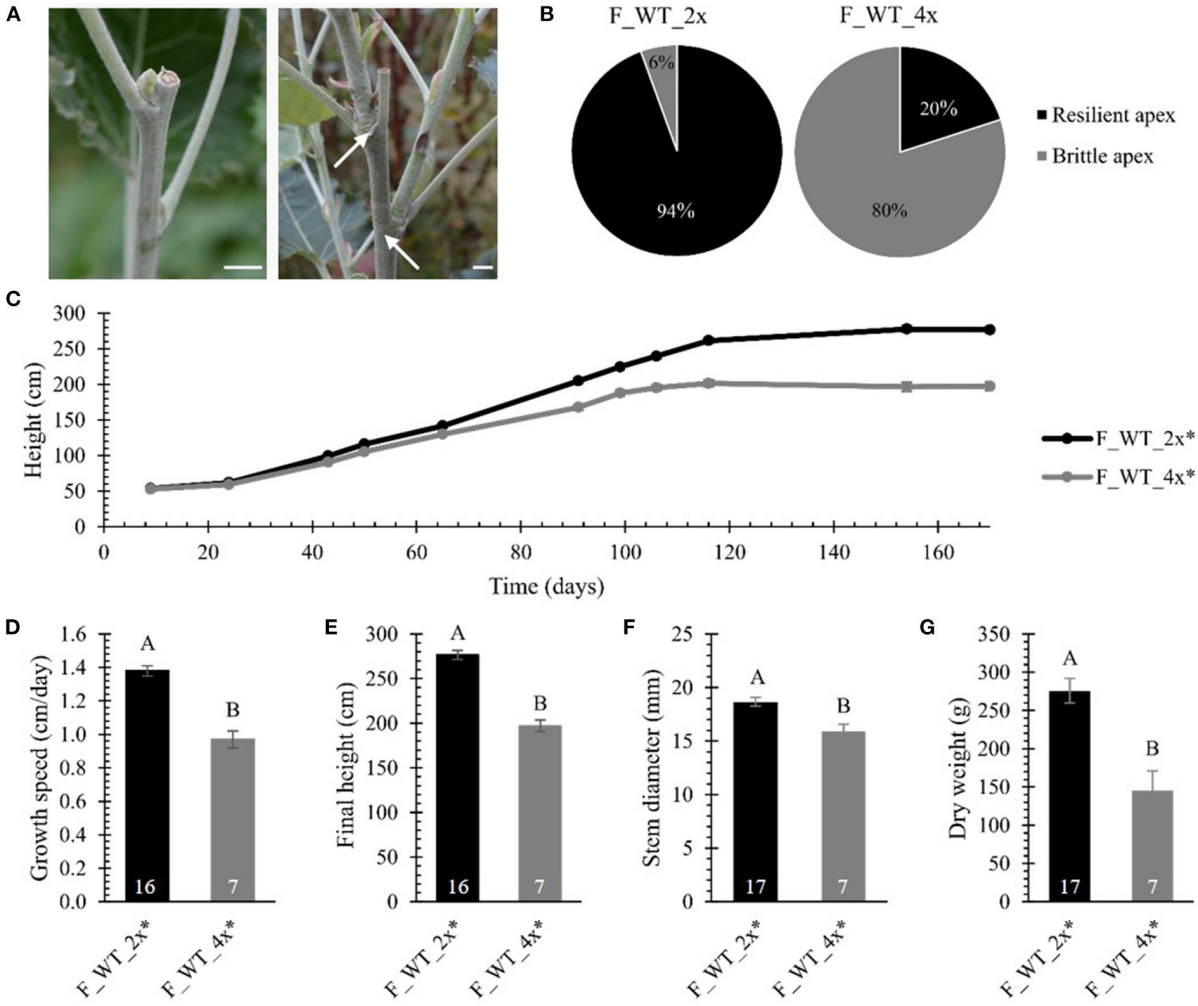
Biomass yield of field-grown di- and tetraploid poplar trees. **(A)** Broken tips of tetraploid poplar trees. Arrows point to axillary buds below the broken tip that grew out, resulting in a bushy phenotype. Scale bars = 1 cm. **(B)** Percentage of poplar trees showing the brittle apex phenotype (day = 170). **(C)** Height over time. The height of the main stem was measured at 11 time points over a period of 170 days. **(D)** Growth speed. **(E)** Final height of the poplars measured at day 170. **(F)** Stem diameter measured at harvest time 50 cm above soil. **(G)** Total dry weight of the debarked tree measured at harvest time. Only field-grown trees that did not develop the brittle apex phenotype were included in the analysis, as specified with the asterisk (*). The number of biological replicates is indicated in the bars. Data shown are adjusted mean ± standard error of the mean (SEM). Different letters represent significant differences at the 0.05 significance level. The significance differences for (C) are provided in Supplementary Table 3. For a detailed description of the models fitted to the data and the statistical tests used, see Supplementary Materials and Methods 1.

Finally, we evaluated biomass yield parameters upon polyploidization in field-grown WT poplars. The biomass production of the field-grown di- and tetraploid WT trees was monitored over a period of 170 days. Strikingly, 50 days after planting, the apex of one of the tetraploid trees broke off 5–15 cm below the apex (Figure 4A). The frequency of tetraploids displaying this fragile or brittle apex phenotype gradually increased over time until 80% (28 trees out of 35) of the tetraploid poplars were affected at the end of the first growth season. The breaks were clean, leaving razor-cut-like edges. As a consequence of the damage, several axillary buds below the broken tip grew out, resulting in a bushy phenotype (Figure 4A). During the same period, only one out of 18 diploid trees (6%) had a broken tip (Figure 4B). This phenotype was never observed for greenhouse-grown trees, implying that some environmental factor(s) caused this phenotype. The emergence of the brittle apex phenotype was not related to the line nor the size of the plant, as it occurred on smaller trees (0.90 m) as well as trees of up to 1.90 m tall. Similarly as the greenhouse-grown trees, to quantify the growth parameters, the different lines of the WT tetraploid trees were combined and termed F_WT_4x. For the growth analyses, only those trees without broken tip were included (17 replicates for F_WT_2x and 7 replicates for F_WT_4x). Consistent with the results obtained from the greenhouse-grown trees, the tetraploid trees showed a yield penalty compared with their diploid counterparts. From day 43 onwards, the height of the main stem of the tetraploid poplars was significantly shorter as a result of polyploidization (Figure 4C; Supplementary Table 3). The growth speed of tetraploids was 29.71% slower and, hence, their final height (at day 170) was 28.69% shorter compared with the diploid trees (Figures 4D,E; Supplementary Table 2). At the time of harvest (January 2017), the stem diameter measured 50 cm above soil level and total dry weight of the debarked trees were reduced by respectively 14.69% and 47.20% in tetraploids vs. diploids (Figures 4F,G; Supplementary Table 2).

Taken together, these data show that polyploidization of WT and *hpCAD* hybrid *P. tremula x P. alba* results in a yield penalty, both when trees were grown in the greenhouse and in the field. In addition, the tetraploid WT poplar trees that were grown in the field were more susceptible to damage caused by environmental factors, as shown by the brittle apex phenotype.

### Biomass composition is altered in field-grown, but not greenhouse-grown, allotetraploid poplars

Both the growth reduction as well as the brittle apex phenotype observed in tetraploid poplars grown in the field hinted toward increased susceptibility to biotic or abiotic stress. However, the tetraploid trees did not show any obvious signs of infection, making an abiotic stress factor such as mechanical stress due to wind or severe weather conditions a more likely cause for the phenotype. Because the mechanical properties of a plant are largely determined by the structure and composition of its cell walls (Horvath et al., 2010; Xi, 2018), a detailed cell wall characterization was performed on debarked stem segments sampled at the basis of the main stems of greenhouse- and field-grown di- and tetraploid trees. First, a crude cell wall extraction with water, ethanol, chloroform and acetone was performed on the dry biomass to prepare the CWR (Table 1). A small, but significant reduction of 1.14% in the amount of CWR was found between greenhouse-grown WT di- and tetraploids, but not between *hpCAD* di- and tetraploids. However, the greenhouse-grown *hpCAD* lines deposited less CWR compared with the WT (a reduction of 3.30% and 2.78% for di- and tetraploids, respectively). Similarly as for the greenhouse-grown trees, the CWR of field-grown WT tetraploid trees was reduced by a mere, albeit significant, 1.84% compared with the diploid field-grown control trees.

**Table 1:** Cell wall composition of di- and tetraploid poplar trees.

	**CWR**		**Cellulose**		**MPS**		**Lignin**	
**Genotype**	**n**	**(% DW)**	**n**	**(% CWR)**	**n**	**(% CWR)**	**n**	**(% CWR)**
GH_WT_2x	8	91.12 ± 0.26 (a)	8	54.18 ± 1.99 (a)	8	33.94 ± 0.97 (a)	7	18.27 ± 0.58 (a,b)
GH_WT_4x	16	90.08 ± 0.19 (b)	16	52.50 ± 1.40 (a)	16	33.86 ± 0.69 (a)	16	18.64 ± 0.38 (a)
GH_*hpCAD*_2x	8	88.12 ± 0.26 (c)	8	51.20 ± 1.99 (a)	7	36.18 ± 1.04 (a)	8	17.50 ± 0.54 (a,b)
GH_*hpCAD*_4x	24	87.58 ± 0.15 (c)	24	52.30 ± 1.15 (a)	24	34.03 ± 0.56 (a)	24	17.08 ± 0.31 (b)
F_WT_2x	9	87.75 ± 0.35 (A)	9	45.25 ± 0.78 (A)	9	41.73 ± 0.76 (A)	9	20.27 ± 0.27 (A)
F_WT_4x	18	86.13 ± 0.25 (B)	17	45.91 ± 0.56 (A)	17	39.69 ± 0.55 (B)	18	22.17 ± 0.19 (B)

The cell wall residue (CWR) was determined gravimetrically after a sequential extraction and is expressed as a percentage of dry weight (DW). Crystalline cellulose was determined with the Updegraff method, matrix polysaccharides (MPS) content was weighed after a TFA extraction and lignin content was analyzed with the Klason method. The amount of cellulose, MPS and lignin are expressed as a percentage of CWR. Data of greenhouse (GH)- and field (F)-grown poplars are separated by a black bar because they were collected in different experiments. The number of biological replicates (n) is indicated. Values given are adjusted mean ± standard error of the mean (SEM). Different letters represent significant differences at the 0.05 significance level. For a detailed description of the models fitted to the data and the statistical tests used, see Supplementary Materials and Methods 1.

Next, the relative amounts of the main cell wall polymers (i.e., cellulose, MPS and lignin) were determined in the different biomass samples (Table 1). No significant differences for any of the cell wall polymers were observed in biomass from greenhouse-grown trees neither between diploids and tetraploids in WT and in *hpCAD* lines, nor between WT and *hpCAD* diploid lines. Only the lignin amount of *hpCAD* tetraploid lines was significantly decreased by 8.35% compared with that of WT tetraploid trees. Under field conditions, again, the cellulose content was not significantly affected as a result of polyploidization. However, field-grown tetraploid WT trees had a 4.88% decrease in MPS and a 9.36% increase in lignin compared with field-grown WT diploids.

Subsequently, the monosaccharide composition of the MPS was determined through analysis of the TFA extract by GC-MS. Xylose was the major constituent of the MPS, regardless of the ploidy level and the growth conditions (Table 2). Greenhouse-grown poplars had a significantly lower amount of MPS-glucose in WT tetraploids (a reduction of 55.58%) and of MPS-xylose in *hpCAD* tetraploids (a reduction of 3.58%) compared with their diploid counterparts. *CAD1*-downregulation, only affected the MPS-glucose levels in the diploid genetic background (a reduction of 63.30%). The arabinose, fucose, galactose, mannose and rhamnose content of the MPS were not significantly different after polyploidization, although a general trend toward higher levels of these sugars was observed in greenhouse-grown WT and *hpCAD* tetraploids. Field-grown WT poplars had a significantly higher MPS-glucose (an increase of 87.39%) and a significantly lower MPS-xylose (a reduction of 3.11%) level in tetraploids compared with diploids. Similar to the MPS composition of the greenhouse-grown WT tetraploid poplars, the amount of the other MPS-sugars of field-grown WT tetraploid poplars did not differ significantly. In general, poplar polyploidization did not seem to affect the MPS composition much, except for the MPS-glucose levels that diminished in tetraploids grown in greenhouse conditions and increased in tetraploids grown in field conditions, and MPS-xylose levels that decreased in tetraploids grown in field conditions.

**Table 2:** Monosaccharide composition of the matrix polysaccharides of di- and tetraploid poplar trees.

**Genotype**	**Arabinose**		**Fucose**		**Galactose**		**Glucose**		**Mannose**		**Rhamnose**		**Xylose**	
	**n**	**(mol%)**	**n**	**(mol%)**	**n**	**(mol%)**	**n**	**(mol%)**	**n**	**(mol%)**	**n**	**(mol%)**	**n**	**(mol%)**
GH_WT_2x	8	0.66 ± 0.29 (a)	8	0.15 ± 0.14 (a)	8	4.55 ± 0.74 (a)	8	3.79 ± 0.67 (a)	8	0.80 ± 0.32 (a)	8	0.92 ± 0.34 (a)	8	89.17 ± 1.10 (a,b)
GH_WT_4x	15	0.86 ± 0.24 (a)	15	0.22 ± 0.12 (a)	16	5.81 ± 0.59 (a)	16	1.66 ± 0.32 (b)	16	1.22 ± 0.27 (a)	16	1.46 ± 0.30 (a)	16	88.79 ± 0.79 (a)
GH_*hpCAD*_2x	8	0.75 ± 0.31 (a)	8	0.16 ± 0.14 (a)	8	4.07 ± 0.70 (a)	8	1.38 ± 0.41 (b)	8	0.77 ± 0.31 (a)	8	0.95 ± 0.34 (a)	8	91.93 ± 0.96 (b)
GH_*hpCAD*_4x	24	0.93 ± 0.20 (a)	22	0.24 ± 0.10 (a)	22	5.71 ± 0.49 (a)	24	1.30 ± 0.23 (b)	23	0.99 ± 0.21 (a)	24	1.34 ± 0.23 (a)	24	88.64 ± 0.65 (a)
F_WT_2x	9	0.58 ± 0.25 (A)	9	0.14 ± 0.12 (A)	8	2.04 ± 0.50 (A)	9	2.38 ± 0.51 (A)	9	0.44 ± 0.22 (A)	9	0.61 ± 0.26 (A)	8	94.22 ± 0.83 (A)
F_WT_4x	18	0.57 ± 0.18 (A)	17	0.10 ± 0.08 (A)	18	2.78 ± 0.39 (A)	18	4.46 ± 0.49 (B)	15	0.33 ± 0.15 (A)	18	0.56 ± 0.18 (A)	16	91.29 ± 0.72 (B)

MPS content was determined gravimetrically after a TFA extraction (%arabinose + %fucose + %galactose + %glucose + %mannose + % rhamnose + %xylose = 100%). Data of greenhouse (GH)- and field (F)-grown poplars are separated by a black bar because they were collected in different experiments. The number biological replicates (n) is indicated. Values given are adjusted mean ± standard error of the mean (SEM). Different letters represent significant differences at the 0.05 significance level. For a detailed description of the models fitted to the data and the statistical tests used, see Supplementary Materials and Methods 1.

Finally, the lignin composition was quantified by thioacidolysis followed by GC-MS (Table 3). The thioacidolysis reaction releases the traditional lignin units (H, G, and S) that are linked by β-*O*−4-ether bonds in the lignin polymer. We found that the induction of polyploidy had no effect on the general lignin composition when trees were grown under greenhouse and field conditions. More specifically, the levels of the G and S units that make up the bulk of the lignin were not significantly different between di- and tetraploids. Besides the traditional lignin units, we also detected trace amounts (<1 mol%) of S aldehydes, characteristic for *CAD1*-downregulation, in lignin of both *hpCAD* diploids and *hpCAD* tetraploids, but not in lignin of WT. However, the levels of S aldehydes that were incorporated in the lignin did not change after polyploidization. The sum of all the released units is a good estimate of the total thioacidolysis yield and, hence, can be used as a measure for the lignin condensation. On the one hand, the total thioacidolysis yield of greenhouse-grown WT diploids and tetraploids was equal, whereas that of greenhouse-grown *hpCAD* tetraploids was significantly reduced by 19.18% compared with their diploid counterparts. On the other hand, the total thioacidolysis yield of field-grown WT tetraploids was significantly reduced by 15.03% compared with their diploid counterparts. The decrease in total thioacidolysis yield of greenhouse-grown *hpCAD* and field-grown WT tetraploids implies that their lignin is more condensed as a result of polyploidization.

**Table 3:** Lignin composition of di- and tetraploid poplar trees.

**Genotype**	**Total**		**Total**		**H**		**G**		**S**		**S aldehyde**		**S/G**	
	**thioacidolysis yield**		**thioacidolysis yield**											
	**n**	**(μmol / mg CWR)**	**n**	**(μmol / mg Klason lignin)**	**n**	**(mol%)**	**n**	**(mol%)**	**n**	**(mol%)**	**n**	**(mol%)**	**n**	**S/G**
GH_WT_2x	6	0.44 ± 0.03 (a)	6	2.28 ± 0.24 (a)	7	1.11 ± 0.40 (a)	6	33.26 ± 1.92 (a)	6	65.49 ± 1.94 (a)	7	n.d.	6	1.97 ± 0.57 (a)
GH_WT_4x	15	0.45 ± 0.02 (a)	16	2.30 ± 0.15 (a)	16	1.32 ± 0.28 (a)	15	34.79 ± 1.23 (a)	15	63.87 ± 1.24 (a)	16	n.d.	15	1.84 ± 0.35 (a)
GH_*hpCAD*_2x	8	0.51 ± 0.02 (a)	8	2.92 ± 0.15 (a)	8	1.12 ± 0.37 (a)	8	37.69 ± 1.71 (a)	8	60.46 ± 1.73 (a)	8	0.73 ± 0.30 (a)	8	1.61 ± 0.45 (a)
GH_*hpCAD*_4x	24	0.40 ± 0.01 (b)	24	2.36 ± 0.09 (b)	22	1.83 ± 0.29 (a)	24	40.32 ± 1.00 (a)	24	57.18 ± 1.01 (a)	24	0.76 ± 0.18 (a)	24	1.42 ± 0.24 (a)
F_WT_2x	7	0.66 ± 0.02 (A)	7	3.23 ± 0.10 (A)	8	1.61 ± 0.44 (A)	8	29.48 ± 1.61 (A)	8	68.91 ± 1.64 (A)	8	n.d.	8	2.34 ± 0.54 (A)
F_WT_4x	17	0.58 ± 0.01 (B)	17	2.61 ± 0.07 (B)	17	1.52 ± 0.29 (A)	17	31.17 ± 1.09 (A)	17	67.31 ± 1.11 (A)	17	n.d.	17	2.16 ± 0.35 (A)

Lignin composition was determined with the thioacidolysis method (%H + %G + %S + %S aldehyde = 100%). The total thioacidolysis yield is the sum of H, G, S and S aldehyde content. Data of greenhouse (GH)-grown WT poplars, GH-grown *hpCAD* poplars and field (F)-grown poplars are separated by a black bar because they were collected in different experiments. The number of biological replicates (n) is indicated. Values given are adjusted mean ± standard error of the mean (SEM). Different letters represent significant differences at the 0.05 significance level. n.d., not detected. For a detailed description of the models fitted to the data and the statistical tests used, see Supplementary Materials and Methods 1.

Taken together, under greenhouse conditions, there is no significant effect on the cellulose, MPS and lignin amount, whereas under field conditions, the cell wall composition was altered upon whole genome duplication. Field-grown tetraploids had less MPS and more lignin than diploids. These data illustrate that there is a clear interaction between polyploidization and the environment on the deposition of cell wall polymers.

### Saccharification efficiency is similar in greenhouse- and field-grown allotetraploid poplars

**Figure 5:**
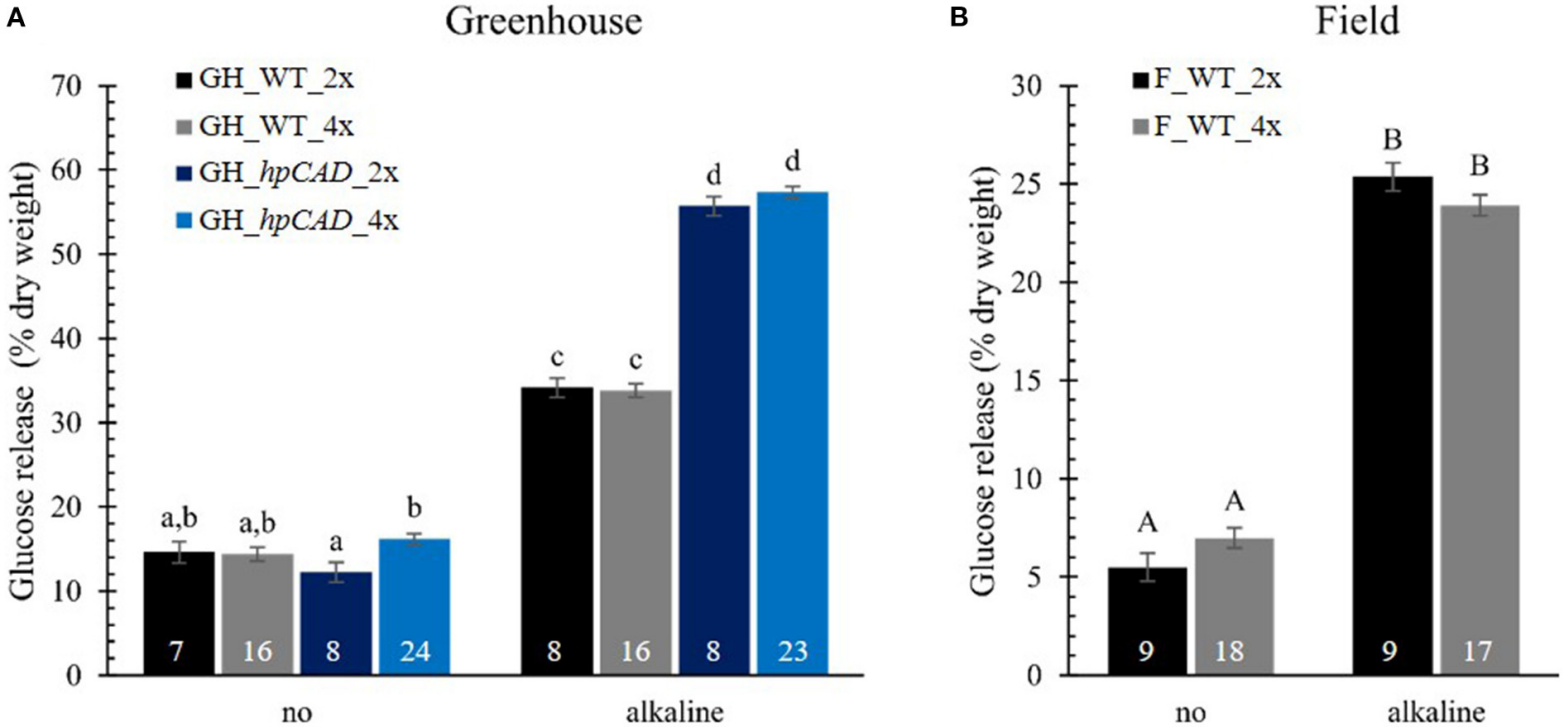
Saccharification yield of di- and tetraploid poplar trees. Glucose release without (no) and with alkaline pretreatment was determined after 10 h and expressed as % of dry weight. Data of greenhouse (GH)-grown **(A)** and field (F)-grown **(B)** poplars were collected in different experiments. The number of biological replicates is indicated in the bars. Data shown are adjusted mean ± standard error of the mean (SEM). Different letters represent significant differences at the 0.05 significance level. For a detailed description of the models fitted to the data and the statistical tests used, see Supplementary Materials and Methods 1.

To evaluate the effect of polyploidy on downstream processing of poplar wood, we performed a saccharification assay and measured the glucose released after 10 h of enzymatic treatment (Figure 5; Supplementary Table 4). Saccharification assays were performed without and with alkaline (NaOH) pretreatment based on the positive effect of the latter on the saccharification yield of *hpCAD* lines (Van Acker et al., 2017). As expected, alkaline pretreatment of the biomass prior to saccharification improved the glucose release for all samples. In general, glucose levels were two- to fivefold higher for biomass pretreated with NaOH compared with biomass that was not pretreated. The positive effect of *CAD1*-downregulation on the saccharification yield after alkaline pretreatment could also be confirmed. Whereas without pretreatment the glucose release of *hpCAD* diploids and tetraploids was equal to that of their non-transgenic counterparts, the cellulose-to-glucose conversion upon alkaline pretreatment increased with 63.11% and 69.54%, respectively. Concerning the effect of polyploidization, no significant differences were observed between WT tetraploid poplars and their respective diploid counterparts, grown in either greenhouse or field conditions and irrespective of the inclusion of a pretreatment. In contrast, we found that greenhouse-grown *hpCAD* tetraploids released 32.43% more glucose without pretreatment, but not with an alkaline pretreatment, compared with greenhouse-grown *hpCAD* diploids.

### Stem anatomy and mechanical properties are largely preserved in WT allotetraploid poplars

**Figure 6:**
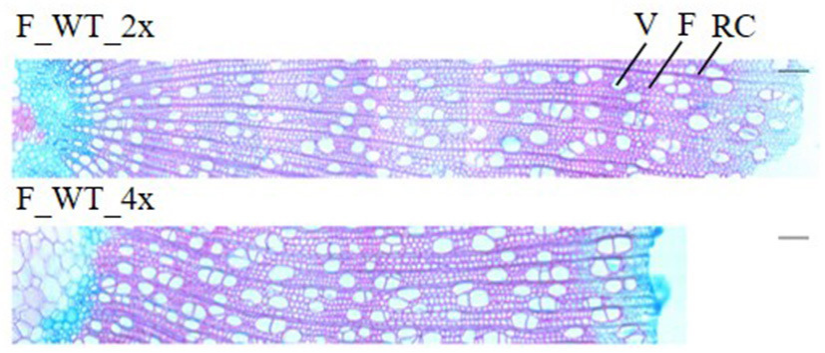
Stem anatomy of field-grown di- and tetraploid poplar trees. The main stem of field-grown WT trees was harvested 1 m above soil level in August 2017 of the second growth season. Transverse stem sections (from pith to bark) stained with astra blue, chrysiodine and acridine red are shown. Pictures are representative for the different independent lines. V, vessel; F, fiber; RC, ray cell. Scale bars = 100 μm.

Field-grown WT tetraploids displayed a brittle apex phenotype. To investigate whether this phenotype is caused by anatomical abnormalities, transversal stem sections were made and triple stained with astra blue, chrysiodine and acridine red (Figure 6). For this, the main stem of di- and tetraploid trees was harvested 1 m above soil level in the second growth season (August 2017). At that time, the tetraploids already showed the brittle apex phenotype. Field-grown di- and tetraploids had an equal vessels to fibers ratio per selected area (Supplementary Table 5). In addition, no significant differences in the average vessel or fiber area per selected area were observed. However, stem sections of the tetraploid poplars showed a 47.98% increase in vessel lumen, i.e., the total vessel area per selected area as compared with the diploid trees. This means that these tetraploids have a higher proportion of cavities present in their stem, potentially weakening the trees when exposed to the environment.

**Figure 7:**
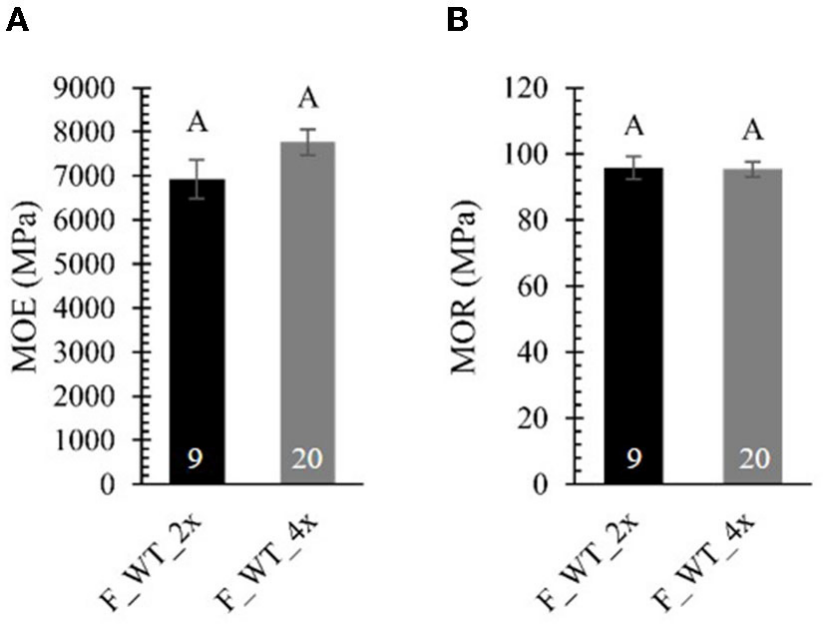
Mechanical strength of field-grown di- and tetraploid poplar trees. Stem samples were subjected to a three-point bending test. From the force-displacement curve, the modulus of elasticity [MOE, **(A)**] and the modulus of rupture [MOR, **(B)**] were calculated. The number of biological replicates is indicated in the bars. Data shown are adjusted mean ± standard error of the mean (SEM). Different letters represent significant differences at the 0.05 significance level. For a detailed description of the models fitted to the data and the statistical tests used, see Supplementary Materials and Methods 1.

To test whether the brittle apex phenotype might be explained by differences in mechanical strength, we performed a three-point bending tests on 4-year-old field-grown di- and tetraploid WT trees (Figure 7; Supplementary Table 6). To be able to make a valid comparison between di- and tetraploids that show a difference in biomass yield, we attempted to harvest stem samples with a comparable diameter for the three-point bending test (ten diploid samples harvested from the top and 20 tetraploid samples harvested from the base of the stem). For these tests, stems were mounted on two supporting pins, whereupon an increasing force was applied to the center *via* a third indenting pin until the sample breaks. A force-displacement curve was plotted for every sample (Supplementary Figure 3). Stem samples of diploids could resist a larger force than those of tetraploids because, despite our efforts to harvest stem samples with a comparable diameter, the stems of the diploid poplars had a larger diameter. We also observed that, in contrast to the diploids (0/10), the tetraploids had a different failure behavior (8/20), visible as sudden decreases in the force-displacement curve. These small cracks before fully breaking might be attributed to a higher brittleness and hint that the field-grown tetraploids are more fragile than the diploids when exposed to the environment (e.g., wind). From the force-displacement curves, two parameters of mechanical strength were calculated, the MOE and the MOR. Although the formulas for both parameters correct for the difference in diameter of di- and tetraploid poplar stems, they assume a perfect solid cylinder as sample, which was not the case for most of our stem samples. The MOE, derived from the elastic region of the curve is a measure for the resistance to non-permanent deformation: elastic materials have a small MOE, whereas stiff materials have a large MOE. The MOR, derived from the plastic region of the curve, is a measure for the bending strength at constant maximal force: materials with a small MOR break faster, whereas materials with a large MOR can withstand a larger force before breaking. No significant differences in the MOE and MOR were found between di- and tetraploid WT poplars grown in the field (Figures 7A,B). As such, the brittle apex phenotype observed in field-grown tetraploids cannot be explained by a difference in mechanical properties, at least not by those parameters deduced from the three-point bending test. However, the sudden decreases in the force-displacement curves of tetraploids do suggest some difference in mechanical properties.

## Discussion

### Whole genome duplication of *P. tremula* x *P. alba* cv. INRA 717-1B4 impairs vegetative growth

To study the effects of whole genome duplication on biomass yield and composition, allotetraploids were generated of hybrid *P. tremula* x *P. alba* cv. INRA 717-1B4, a model clone frequently used in tree biotechnology research. Under both greenhouse and field conditions, tetraploid poplar trees grew more slowly and had a significant yield penalty compared with their corresponding diploid counterparts. A similar slow vegetative growth has been observed in tetraploid poplars obtained after chromosome doubling of a diploid hybrid progeny (*P. pseudo-simonii* × *P. nigra* Zheyin3#) × (*P*. × *beijingensis*) and could be attributed to the upregulation of growth-promoting auxin- and gibberellin-related miRNAs and the downregulation of senescence-related miRNAs (Xu et al., 2020). This opposite trend in the expression of these two classes of differentially expressed genes decreased the photosynthetic rate, explaining the observed yield penalty (Xu et al., 2020).

The negative impact of whole genome duplication on growth speed and plant height is not limited to poplar, but has also been observed in empress tree, Rangpur lime, birch, acacia, willow and apple tree (Tang et al., 2010; Allario et al., 2011; Mu et al., 2012; Griffin et al., 2015; Dudits et al., 2016; Ma et al., 2016; Hias et al., 2017). Interestingly, in birch and willow, the reduction in growth speed and height was accompanied by a significant increase in stem diameter, resulting in an increase in total biomass (Mu et al., 2012; Dudits et al., 2016). We did not observe a similar compensation effect in the tetraploid poplar lines, indicating that the effect of whole genome duplication on plant growth is species- and genotype-dependent. On the other hand, the phenotypic differences between poplar and the other tetraploid woody species might find their origin in the nature of the parental line(s) used for tetraploidization, i.e. whether the tetraploid was derived from an intra- or interspecific hybrid. Indeed, whole genome duplication of *P. tremula* x *P. alba* cv. INRA 717-1B4 and (*P. pseudo-simonii* × *P. nigra* Zheyin3#) × (*P*. × *beijingensis*) resulted in allotetraploids, whereas autotetraploids were obtained in the case studies focusing on birch or willow. More systematic studies involving tetraploidization of pure and hybrid trees are needed to support or refute this hypothesis.

### Whole genome duplication of *P. tremula* x *P. alba* cv. INRA 717-1B4 affects biomass composition of field-grown WT tetraploids and causes a brittle apex phenotype

In *Arabidopsis thaliana*, ploidy level is negatively correlated with lignin content i.e., the higher the ploidy level, the lower the lignin content (Corneillie et al., 2019). Given that lignin is one of the main factors causing lignocellulosic biomass recalcitrance to enzymatic hydrolysis (Chen and Dixon, 2007; Van Acker et al., 2013; Zoghlami and Paës, 2019), the saccharification yield of low-lignin tetraploid Arabidopsis plants was higher than that of diploids (Corneillie et al., 2019). In contrast to Arabidopsis, the biomass composition (i.e. cellulose, MPS and lignin amount) of the greenhouse-grown di- and tetraploid WT and *CAD1*-downregulated poplar analyzed here was not different. Accordingly, also the saccharification yield remained equal, except for the small increase in glucose release observed in saccharifications without biomass pretreatment of tetraploid *hpCAD* poplars. Interestingly, a shift in biomass composition was observed for field-grown tetraploid poplars: the lignin content increased by 9.36% at the expense of MPS. In addition, the total thioacidolysis yield was reduced in field-grown—but not in greenhouse-grown—WT tetraploids in comparison with their diploid counterparts, implying that the lignin polymer is more condensed when tetraploids are grown in the field. However, this shift in lignin amount and condensation degree in field-grown tetraploid poplars did not translate into a difference in saccharification yield, regardless of a pretreatment. In contrast to our allotetraploid poplars, field-grown autotetraploid willows had a lower lignin content than diploids (Serapiglia et al., 2015), and for autotetraploid acacia, less alkali was consumed to yield similar amounts of Kraft pulp as diploids, indicating an increased biomass processing efficiency (Griffin et al., 2014). Again, these different findings indicate that the effect of whole genome duplication is not easily predictable, and most likely species- and genotype-dependent.

When grown in the field, the tetraploid *P. tremula x P. alba* cv. INRA 717 1B4 trees developed a brittle apex phenotype. Interestingly, some old studies on petunia and cassava mention a similar brittle phenotype upon whole genome duplication. In the case of petunia, tetraploids were described as “rather ugly plants, which were very fragile because after a storm great parts of the tetraploid material was broken, whereas the diploids were hardly damaged” (Levan, 1939), and tetraploid cassava plants were found to be “less vigorous, and their stem, although stouter is less hardy and more brittle” (Abraham et al., 1964). The fact that the brittle apex phenotype in our study was only present under field conditions makes it tempting to link the phenotype to the observed increase in lignin content in these plants, making them more sensitive to suboptimal growth conditions. A similar association between lignin content and brittleness was made before. For example, the *brittle stalk2* maize mutant, named after the fact that its aerial parts are easily broken in the field as a result of wind damage, has an enriched lignin content and a reduced mechanical strength compared with WT plants (Ching et al., 2006; Sindhu et al., 2007). Similarly, a brittle wood phenotype has been reported for *P. trichocarpa* downregulated in glycosyltransferase *8D1* and *8D2* genes implicated in xylan biosynthesis. The stem of this double mutant had a reduced modulus of elasticity (MOE) and modulus of rupture (MOR), which was correlated with a decrease in xylan content and an increase in lignin quantity (Li et al., 2011). Inversely, both in Arabidopsis and poplar, downregulation of lignin biosynthesis reduces cell wall stiffness as reflected in a decrease of the MOE (Köhler and Spatz, 2002; Koehler and Telewski, 2006; Bjurhager et al., 2010; Özparpucu et al., 2017; De Meester et al., 2018). Together, these data demonstrate that lignin plays an important role in cell wall stiffness. However, despite the increase in lignin content, no major differences in mechanical properties could be demonstrated between field-grown di- and tetraploid poplars. This could be a direct consequence of some technical limitations when performing the three-point bending test used to calculate MOE and MOR parameters. The three-point bending test and related calculations as performed here, require a sample that is a perfect solid cylinder for optimal performance. Our samples did not meet this criterion, because they contained the central pith and had a moisture content of 6%. In addition, in an attempt to analyze stem segments with a comparable diameter, diploid stem samples were harvested from the top of the stem, whereas tetraploid plants had to be sampled at the base of the stem. As such, the number and thickness of the year rings were also different between di- and tetraploid samples. Given all these limitations, the comparison of the obtained absolute values for MOE and MOR with literature should be made with caution. Nonetheless, the same order of magnitude was found by Horvath et al. (2010).

### The environment has a strong effect on the biomass yield and composition of *hpCAD* poplar, whereas whole genome duplication does not

The diploid *hpCAD* line showed a biomass yield penalty, but no shift in lignin content compared with its WT counterpart when grown in the greenhouse. Similar results were obtained when this *hpCAD* line was grown for 3 years in the field (De Meester et al., 2022), whereas in another study no yield penalty and a 10% decrease in lignin content was obtained when this *hpCAD* line was grown under greenhouse conditions (Van Acker et al., 2017). Independent of these contradictory results, all these studies support a link between growth speed and lignin content in *CAD1*-downregulated poplars. Because plant growth and lignin biosynthesis compete for the same carbon resources, it is conceivable that when growth is slowed down, plants have more time to fully lignify their cell walls. Or vice versa, that a restored flux toward lignin has a negative impact on pant growth and, hence, on biomass yield. The dissimilarity in biomass yield and lignin content between the studies are likely due to differences in environmental factors such as irrigation frequency and temperature. An example illustrating this aspect even more profoundly is that of the *Medicago truncatula CAD* loss-of-function mutant. The plants appeared normal under standard conditions in the greenhouse, but were dwarfed when grown at 30°C (Zhao et al., 2013). Together, these studies demonstrate that caution is required when interpreting, comparing or extrapolating published results from lignin mutants, because the environment might influence biomass yield and composition, as also discussed by De Meester et al. (2022).

The generated tetraploid *hpCAD* poplars displayed a uniform red coloration of the xylem, incorporated sinapaldehydes into their lignin and had an increased saccharification yield upon alkaline pretreatment. These features have been described before for *CAD1*-downregulated lines and indicate that silencing of *CAD1* was maintained upon whole genome duplication. Similar to the greenhouse-grown WT poplars, chromosome doubling of *hpCAD* had a negative effect on biomass yield, but did not affect its composition. However, despite the absence of an effect on lignin content, saccharification yield without pretreatment slightly improved upon polyploidization.

### *P. tremula* x *P. alba* cv. INRA 717-1B4 tetraploids may serve as parental germplasm to generate triploids

Triploid breeding is an excellent approach to improve lignocellulosic biomass yield and composition in shrub willow and poplar (Serapiglia et al., 2014, 2015; Kang, 2016). The surface area of poplar triploid leaves, as a consequence of cell size enlargement, is significantly larger than that of diploids (Nilsson-Ehle, 1936a; Zhang et al., 2019). The formation of these giant leaves with superior photosynthetic efficiency could be attributed to the upregulation of growth hormone-related and chlorophyll biosynthesis genes (Du et al., 2019; Wu et al., 2021). Moreover, most lignin biosynthesis genes are downregulated in triploid leaves compared with diploid leaves (Xu et al., 2022). Although biomass traits of some triploids are excellent, some may also have adverse characteristics, such as a low wood basic density and the potential to be easily broken by wind after planting. For this reason, allotriploids of *P. tremula* × *P. tremuloides* have been eliminated from most breeding programs (Kang, 2016).

Crossing diploid and tetraploid parents, i.e., hybridizing n with 2n gametes, is historically the most economic and fastest practice for triploid production (Ulrich and Ewald, 2014). A beneficial effect on biomass yield and composition has already been shown for triploids of *P. tremula* and *P. alba*, respectively. More specifically, in nature, a giant form of *P. tremula* was discovered with increased stem height, diameter and volume, which turned out to be a spontaneous triploid (Müntzing, 1936; Nilsson-Ehle, 1936b). And, comparative trials with a range of allotriploid clones involving *P. alba* showed that the stem volume and cellulose content were higher and the lignin content lower than those of the diploid controls (Kang, 2016). As such, combining both species like in the allotetraploid WT *P. tremula* x *P. alba* cv. INRA 717-1B4 generated in this study, may provide an important female parental germplasm for generating triploid poplars in the future. In addition, such crosses would allow investigating whether the reduction in biomass yield and the brittle apex phenotype of allotetraploid WT *P. tremula* x *P. alba* cv. INRA 717-1B4 are maintained in the triploids. Nevertheless, given the fact that the triploid offspring will all be genetically different, there is no reason to assume that this would be the case for all triploids when screening a large population.

In conclusion, we showed that combining polyploidy and heterosis in WT and *hpCAD* poplar did not result in a higher biomass yield, nor in an improved biomass composition for saccharification. Furthermore, we demonstrated that the outcome of a whole genome duplication is strongly dependent on the environment, highlighting the importance of field trials.

## Data availability statement

The original contributions presented in the study are included in the article/Supplementary material, further inquiries can be directed to the corresponding author.

## Author contributions

MW analyzed the data and wrote the article. SC designed and performed the experiments. JVD created the set of tetraploid poplars. AD performed the chromosome spreads. JVA and JVdB performed the three-point bending test and interpreted these results at UGent-Woodlab. BV and WB conceived the project, supervised the experiments and helped in writing the article. All authors contributed to the article and approved the submitted version.

## Funding

This work was supported by grants from the Multidisciplinary Research Partnership Biotechnology for a Sustainable Economy (01MRB510W) of Ghent University. MW and SC were supported by the Research Foundation Flanders (FWO) for a predoctoral fellowship under project numbers 3S019619 and 3G032912, respectively. WB is indebted to the Energy Transition Funds project AdLibio and Adv Bio.

## Conflict of interest

The authors declare that the research was conducted in the absence of any commercial or financial relationships that could be construed as a potential conflict of interest.

## Publisher's note

All claims expressed in this article are solely those of the authors and do not necessarily represent those of their affiliated organizations, or those of the publisher, the editors and the reviewers. Any product that may be evaluated in this article, or claim that may be made by its manufacturer, is not guaranteed or endorsed by the publisher.

## Abbreviations

2x: diploid
4x: tertraploid
CWR: cell wall residue
GC-MS: gas chromatography-mass spectrometry
*hpCAD*: hairpin-downregulated *CINNAMYL ALCOHOL DEHYDROGENASE1*
MOE: modulus of elasticity
MOR: modulus of rupture
MPS: matrix polysaccharides
TFA: trifluoroacetic acid
WT: wild type.
