# Supplementary material for: Whole genome duplication of wild-type and CINNAMYL ALCOHOL DEHYDROGENASE1-downregulated hybrid poplar reduces biomass yield and causes a brittle apex phenotype in field-grown wild types
Source: Front Plant Sci. 2022 Sep 9;13:995402. doi: 10.3389/fpls.2022.995402 (PMC9504066; doi:10.3389/fpls.2022.995402)
Supplement: Supplementary file 1 [file Data_Sheet_1.docx]

Supplementary Material

# Supplementary Materials and Methods

**Supplementary Materials and Methods 1:** Detailed description of the models fitted to the greenhouse and field data and the statistical tests used.

The response variable (y) plant height of field-grown trees was measured at multiple time points and analyzed as repeated measurements using residual maximum likelihood (REML) variant component analysis, as implemented in GenStat (version 21.1). Briefly, **Model 1** is a linear mixed model (LMM) of the following form (random terms underlined): y = T_0_ + block + genotype + time + genotype × time + block × time + tree × time and was fitted to the repeated measurements. The covariate T_0_ accounts for differences in height at the start of the measurements. The term tree × time represents the residual error term with dependent errors (i.e. the repeated measurements are taken on the same tree, causing correlations among observations). Several covariance models were fitted to the data to account for the correlation present in the data. The antedependence correlation model of order 1 (ANTE1) was finally selected as best fitted model based on the Akaike’s information criterion coefficient. The ANTE covariance model assumes that correlation between observations decays as the measurements are collected further apart in time, just like the autoregressive covariance model does, but allows for changes in correlation structure over time. The first order refers to the fact that recent (t-1) observations affect the current observations made at time point t. The model fitted to the plant height repeated measurements data of greenhouse-grown trees is similar to **Model 1**, except that it does not contain the fixed term block and its interaction term block × time. Observations with a standardized residual larger than three (in absolute value) were considered as outliers and removed from the data. The significances of the fixed terms in **Model 1** and significances of changes between genotype effects over time were assessed using an F-test, as implemented in GenStat (version 21.1). *Post-hoc* comparisons between genotypes at each time point were assessed based on a *t*-test at the 5% significance level.

The response variable (y) growth speed of field-grown trees was analyzed using ANCOVA covariant analysis, as implemented in R (version 3.6.3). Briefly, **Model 2** is a linear model (LM) of the following form: y = µ + T_0_ + block + genotype + error and was fitted to the data. The covariate T_0_ accounts for the differences in height at the start of the measurements. The model fitted to the growth speed data of greenhouse-grown trees is similar to **Model 2**, except that it does not contain the fixed term block. Observations with a standardized residual larger than three (in absolute value) were considered as outliers and removed from the data. The significance(s) of the fixed term(s) in **Model 2** was (were) assessed using an F-test, as implemented in R (version 3.6.3). *Post-hoc* comparisons between genotypes were based on Fisher’s Protected Least Significant Differences (LSD) at the 5% significance level.

The response variables (y) final height, stem diameter, dry weight, CWR, cellulose, MPS, lignin, total thioacidolysis yield, vessel area, fiber area, MOE and MOR of field-grown trees were analyzed using ANOVA variant analysis, as implemented in R (version 3.6.3). Briefly, **Model 3** is a linear model (LM) of the following form: y = µ + block + genotype + error and was fitted to the data. The model fitted to the response variables of greenhouse-grown trees is similar to **Model 3**, except that it does not contain the fixed term block. Observations with a standardized residual larger than three (in absolute value) were considered as outliers and removed from the data. The significance(s) of the fixed term(s) in **Model 3** was (were) assessed using an F-test, as implemented in R (version 3.6.3). *Post-hoc* comparisons between genotypes were based on Fisher’s Protected Least Significant Differences (LSD) at the 5% significance level.

The response variables (y) H, G, S, arabinose, fucose, galactose, glucose, fucose, rhamnose, xylose and vessel lumen of field-grown trees were analyzed in R (version 3.6.3). Briefly, **Model 4** is a generalized linear model (GLM) of the following form: y = µ + block + genotype + error and was fitted to the proportion data with a logit link function. The model fitted to the response variables of greenhouse-grown trees is similar to **Model 4**, except that it does not contain the fixed term block. Observations with a standardized residual larger than three (in absolute value) were considered as outliers and removed from the data. The dispersion parameter was fixed at 1. The significance of the regression was assessed by comparing its deviance with χ2. The significance of genotype effects (on the logit transformed scale) was assessed by pairwise comparisons to the reference level using Z-statistics at the 5% significance level.

The response variables (y) S/G, no vessels / no fibers of field-grown trees were analyzed in R (version 3.6.3). Briefly, **Model 5** is a generalized linear model (GLM) of the following form: y = µ + block + genotype + error and was fitted to the count data with a log link function. The model fitted to the response variables of greenhouse-grown trees is similar to **Model 5**, except that it does not contain the fixed term block. Observations with a standardized residual larger than three (in absolute value) were considered as outliers and removed from the data. The dispersion parameter was fixed at 1. The significance of the regression was assessed by comparing its deviance with χ2. The significance of genotype effects (on the logit transformed scale) was assessed by pairwise comparisons to the reference level using Z-statistics at the 5% significance level.

The response variable (y) glucose release of field-grown trees was analyzed using ANOVA variant analysis, as implemented in GenStat (version 21.1). Briefly, **Model 6** is a linear model (LM) of the following form: y = µ + block_time + genotype + treatment + genotype × treatment + error and was fitted to the data. The fixed term block_time combines the three blocks and the two time points of measurement, resulting in a total of four levels. The model fitted to the glucose release of greenhouse-grown trees is similar to **Model 6**, except that the combined factor block_time was replaced by time having only two levels. Observations with a standardized residual larger than three (in absolute value) were considered as outliers and removed from the data. The significance(s) of the fixed term(s) in **Model 6** was (were) assessed using an F-test, as implemented in GenStat (version 21.1). *Post-hoc* comparisons between genotype by treatment means were based on Fisher’s Protected Least Significant Differences (LSD) at the 5% significance level.

# Supplementary Figures and Tables

## Supplementary Figures

**
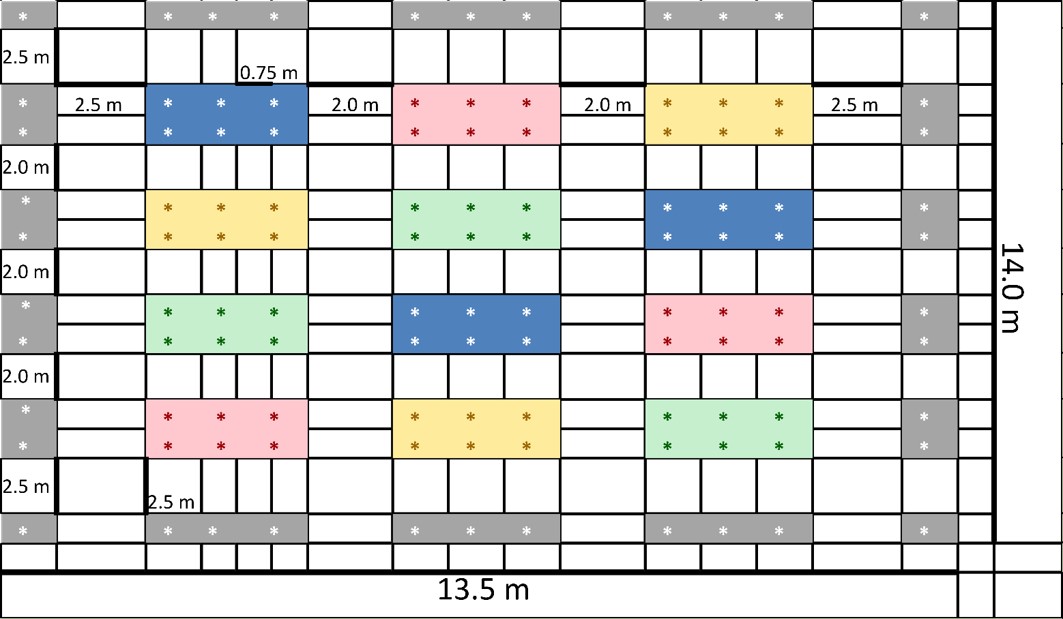
**

**Supplementary Figure 1:** Field trial set-up. The field trial consists of three randomized blocks each containing six clonal replicates per line (F_WT_2x: blue, F_WT_4x line 1: yellow, F_WT_4x line 2: green, F_WT_4x line 3: red) and is surrounded by a border of WT diploid poplar trees (marked in gray). Distances are as indicated.


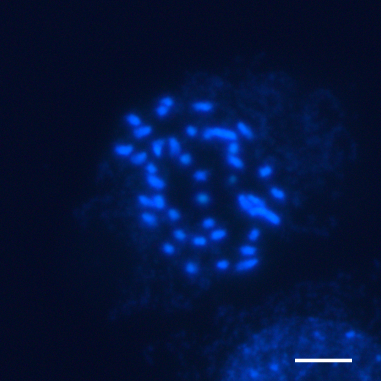

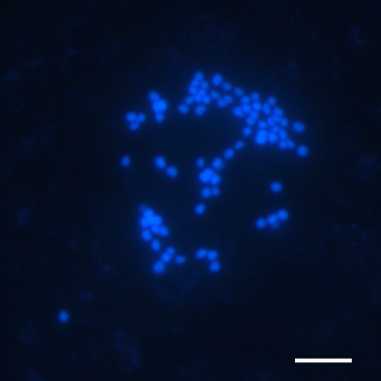

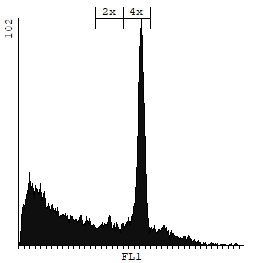

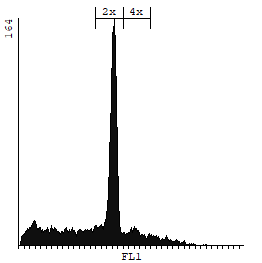


A

B

C

D

Channel (DAPI)

Number of cells

Number of cells

Channel (DAPI)

**Supplementary Figure 2:** Confirmation of the ploidy level of *hpCAD* poplar plantlets grown from colchicine-treated axillary buds via flow cytometry and chromosome spreads. **(A, C)** *hpCAD* diploid poplar. **(B, D)** *hpCAD* tetraploid poplar. Pictures are representative for the different independent tetraploid lines. Scale bars = 5 µm.

**
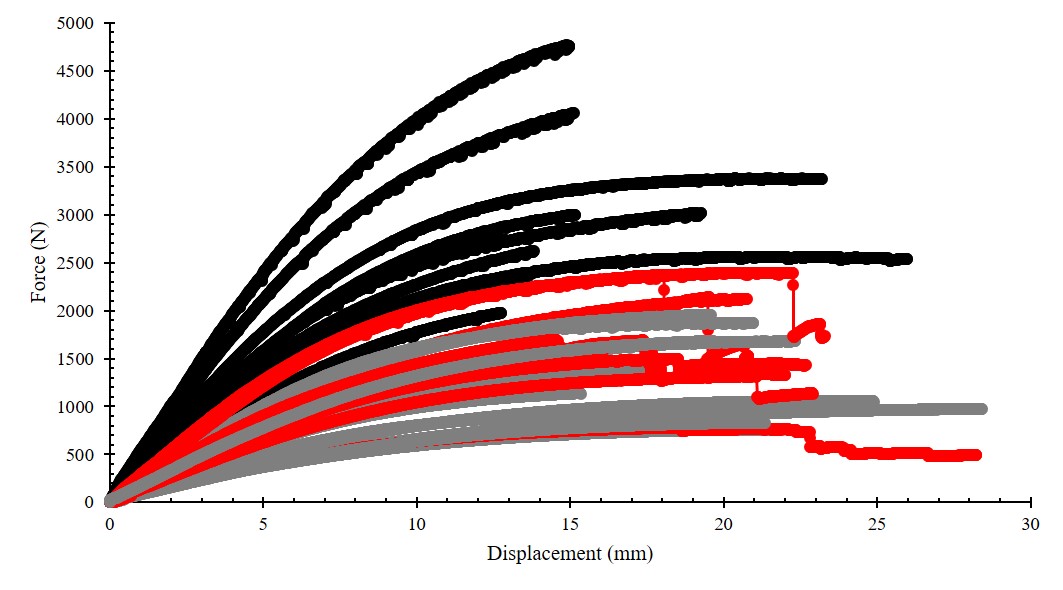
**

**Supplementary Figure 3:** Mechanical properties of field-grown di- and tetraploid poplar trees. Stem samples were subjected to a three-point bending test. A force-displacement curve was plotted for every sample. Stem samples of diploids could resist a larger force than those of tetraploids because, the stems of the diploid poplars had a larger diameter. WT tetraploids displayed a different failure behavior (visible as sudden decreases in the force-displacement curve) compared with WT diploids. F_WT_2x: black (n = 10), F_WT_4x without cracks (n = 12): gray, F_WT_4x with cracks (n = 8): red, with n the number of biological replicates.

## Supplementary Tables

**Supplementary Table 1:** Height over time of greenhouse-grown di- and tetraploid poplar trees. The height of the main stem was measured on a weekly basis over a period of 120 days. The number of biological replicates (n) is indicated. Values given are adjusted mean ± standard error of the mean (SEM). Different letters represent significant differences at the 0.05 significance level. For a detailed description of the models fitted to the data and the statistical tests used, see Supplementary Materials and Methods 1.

| **Genotype** |  | **Height (cm)** | | | | | | | | | | | | | | |  |
| --- | --- | --- | --- | --- | --- | --- | --- | --- | --- | --- | --- | --- | --- | --- | --- | --- | --- |
|  | **n** | **day 21** | **day 28** | **day 36** | **day 42** | **day 49** | **day 56** | **day 63** | **day 70** | **day 77** | **day 85** | **day 91** | **day 99** | **day 105** | **day 114** | **day 120** | |
| GH_WT_2x | 15 | 17.75 ± 4.78 (a) | 23.69 ± 4.78 (a) | 34.22 ± 4.78 (a) | 44.22 ± 4.78 (a) | 56.95 ± 4.78 (a) | 70.95 ± 4.78 (a) | 83.35 ± 4.78 (a) | 99.09 ± 4.78 (a) | 115.02 ± 4.78 (a) | 135.75 ± 4.78 (a) | 153.35 ± 4.78 (a) | 174.02 ± 4.78 (a) | 184.82 ± 4.78 (a) | 202.89 ± 4.78 (a) | 218.89 ± 4.78 (a) | |
| GH_WT_4x | 30 | 14.09 ± 3.46 (a) | 19.45 ± 3.46 (a) | 28.05 ± 3.46 (a) | 37.15 ± 3.46 (a) | 48.12 ± 3.46 (a,b) | 59.82 ± 3.46 (a,b) | 70.22 ± 3.46 (b) | 83.39 ± 3.46 (b,c) | 98.19 ± 3.46 (b,c) | 118.52 ± 3.46 (b,c) | 131.29 ± 3.46 (b) | 148.52 ± 3.46 (b) | 157.52 ± 3.46 (b) | 173.92 ± 3.46 (b) | 185.82 ± 3.46 (b) | |
| GH_*hpCAD*_2x | 15 | 16,21 ± 4.76 (a) | 19.91 ± 4.76 (a) | 26.71 ± 4.76 (a) | 35.24 ± 4.76 (a) | 46.71 ± 4.76 (a,b) | 58.71 ± 4.76 (a,b) | 67.57 ± 4.76 (b) | 86.04 ± 4.76 (a,c) | 103.17 ± 4.76 (a,c) | 124.37 ± 4.76 (a,c) | 138.57 ± 4.76 (b) | 155.11 ± 4.76 (b) | 167.84 ± 4.76 (b) | 187.17 ± 4.76 (c) | 199.51 ± 4.76 (c) | |
| GH_*hpCAD*_4x | 43 | 14.61 ± 2.93 (a) | 19.20 ± 2.93 (a) | 26.50 ± 2.93 (a) | 34.34 ± 2.93 (a) | 43.92 ± 2.93 (b) | 54.66 ± 2.93 (b) | 67.43 ± 2.93 (b) | 77.62 ± 2.93 (b,c) | 90.94 ± 2.93 (b) | 109.34 ± 2.93 (b) | 120.85 ± 2.93 (c) | 136.59 ± 2.93 (c) | 144.87 ± 2.93 (c) | 161.29 ± 2.93 (d) | 167.57 ± 2.93 (d) | |

**Supplementary Table 2:** Biomass yield of di- and tetraploid poplar trees. The growth speed, the final height, the stem diameter 50 cm above soil level and the total dry weight of the debarked tree were determined for every poplar tree. Data of greenhouse (GH)- and field (F)-grown poplars are separated by a black bar because they were collected in different experiments. Only field-grown trees that did not develop the brittle apex phenotype were included in the analyses, as specified with the asterisk (*). The number of biological replicates (n) is indicated. Values given are adjusted mean ± standard error of the mean (SEM). Different letters represent significant differences at the 0.05 significance level. For a detailed description of the models fitted to the data and the statistical tests used, see Supplementary Materials and Methods 1.

|  | **Growth speed** | | **Final height** | | **Stem diameter** | | **Dry weight** | |
| --- | --- | --- | --- | --- | --- | --- | --- | --- |
| **Genotype** | **n** | **(cm/day)** | **n** | **(cm)** | **n** | **(mm)** | **n** | **(g)** |
| GH_WT_2x | 15 | 2.03 ± 0.07 (a) | 15 | 218.89 ± 4.76 (a) | 15 | 10.36 ± 0.46 (a) | 15 | 33.34 ± 2.14 (a) |
| GH_WT_4x | 30 | 1.73 ± 0.05 (b) | 30 | 185.82 ± 3.46 (b) | 30 | 8.78 ± 0.32 (b) | 30 | 16.49 ± 1.52 (b,c) |
| GH_*hpCAD*_2x | 15 | 1.85 ± 0.07 (a,b) | 15 | 199.51 ± 4.76 (c) | 14 | 9.28 ± 0.47 (a,b) | 15 | 20.62 ± 2.14 (b) |
| GH_*hpCAD*_4x | 43 | 1.55 ± 0.04 (c) | 43 | 167.57 ± 2.93 (d) | 44 | 7.47 ± 0.27 (c) | 44 | 13.09 ± 1.25 (c) |
| F_WT_2x* | 16 | 1.38 ± 0.03 (A) | 16 | 276.66 ± 4.99 (A) | 17 | 18.65 ± 0.42 (A) | 17 | 275.60 ± 16.08 (A) |
| F_WT_4x* | 7 | 0.97 ± 0.05 (B) | 7 | 197.29 ± 6.34 (B) | 7 | 15.91 ± 0.66 (B) | 7 | 145.51 ± 25.27 (B) |

**Supplementary Table 3:** Height over time of field-grown di- and tetraploid poplar trees. The height of the main stem was measured at 11 time points over a period of 170 days. Only field-grown trees that did not develop the brittle apex phenotype were included in the analysis, as specified with the asterisk (*). The number of biological replicates (n) is indicated. Values given are adjusted mean ± standard error of the mean (SEM). Different letters represent significant differences at the 0.05 significance level. For a detailed description of the models fitted to the data and the statistical tests used, see Supplementary Materials and Methods 1.

|  | **Height (cm)** | | | | | | | | | | | | | | | | | | | | | |
| --- | --- | --- | --- | --- | --- | --- | --- | --- | --- | --- | --- | --- | --- | --- | --- | --- | --- | --- | --- | --- | --- | --- |
| **Genotype** | **n** | **day 9** | **n** | **day 24** | **n** | **day 43** | **n** | **day 50** | **n** | **day 65** | **n** | **day 91** | **n** | **day 99** | **n** | **day 106** | **n** | **day 116** | **n** | **day 154** | **n** | **day 170** |
| F_WT_2x* | 17 | 53.57 ± 0.35 (A) | 17 | 61.87 ± 1.63 (A) | 17 | 99.17 ± 1.86 (A) | 17 | 115.94 ± 2.32 (A) | 17 | 141.59 ± 3.29 (A) | 16 | 204.78 ± 3.54 (A) | 16 | 224.44 ± 3.71 (A) | 16 | 239.44 ± 3.61 (A) | 16 | 261.27 ± 4.09 (A) | 16 | 277.61 ± 4.96 (A) | 16 | 276.66 ±  4.99 (A) |
| F_WT_4x* | 35 | 52.72 ± 0.22 (A) | 35 | 59.15 ± 1.13 (A) | 35 | 90.27 ± 1.30 (B) | 34 | 105.23 ± 1.63 (B) | 33 | 129.86 ± 2.32 (B) | 13 | 167.73 ± 3.87 (B) | 10 | 187.72 ± 4.50 (B) | 7 | 195.24 ± 4.42 (B) | **7** | 201.35 ± 5.12 (B) | 7 | 196.57 ±  6.28 (B) | 7 | 197.29 ±  6.34 (B) |

**Supplementary Table 4:** Saccharification yield of di- and tetraploid poplar trees. Glucose release without and with alkaline pretreatment was determined after 10 h. Data of greenhouse (GH)- and field (F)-grown poplars are separated by a black bar because they were collected in different experiments. The number of biological replicates (n) is indicated. Values given are adjusted mean ± standard error of the mean (SEM). Different letters represent significant differences at the 0.05 significance level. For a detailed description of the models fitted to the data and the statistical tests used, see Supplementary Materials and Methods 1.

|  | **Without pretreatment** | | **Alkaline pretreatment** | |
| --- | --- | --- | --- | --- |
| **Genotype** | **n** | **Glucose (% dry weight)** | **n** | **Glucose (% dry weight)** |
| GH_WT_2x | 7 | 14.62 ± 1.25 (a,b) | 8 | 34.14 ± 1.17 (c) |
| GH_WT_4x | 16 | 14.39 ± 0.83 (a,b) | 16 | 33.82 ± 0.83 (c) |
| GH_*hpCAD* 2x | 8 | 12.22 ± 1.17 (a) | 8 | 55.68 ± 1.17 (d) |
| GH_*hpCAD* 4x | 24 | 16.18 ± 0.67 (b) | 23 | 57.33 ± 0.69 (d) |
| F_WT_2x | 9 | 5.49 ± 0.72 (A) | 9 | 25.37 ± 0.72 (B) |
| F_WT_4x | 18 | 6.99 ± 0.51 (A) | 17 | 23.91 ± 0.53 (B) |

**Supplementary Table 5:** Stem anatomy of field-grown di- and tetraploid poplar trees. The number and area of vessels and fibers per selected area were determined. The average number of vessels was divided by average the number of fiber cells to provide a ratio. The proportion of vessel lumen is defined as the total vessel area per selected area. The number of biological replicates (n) is indicated, multiple sections per replicate were analyzed as mentioned between brackets. Values given are adjusted mean ± standard error of the mean (SEM). Different letters represent significant differences at the 0.05 significance level. For a detailed description of the models fitted to the data and the statistical tests used, see Supplementary Materials and Methods 1.

| **Genotype** | **n** | **No vessels / No fibers** | **Vessel area (µm^2^)** | **Fiber area (µm^2^)** | **Vessel lumen (%)** |
| --- | --- | --- | --- | --- | --- |
| F_WT_2x | 1 (7) | 0.04 ± 0.08 (A) | 4215.67 ± 276.06 (A) | 208.14 ± 13.47 (A) | 16.86 ± 1.42 (A) |
| F_WT_4x | 4 (24) | 0.07 ± 0.05 (A) | 4641.58 ± 149.09 (A) | 200.12 ± 7.27 (A) | 24.95 ± 0.88 (B) |

**Supplementary Table 6:** Mechanical properties of field-grown di- and tetraploid poplar trees. Stem samples were subjected to a three-point bending test. From the force-displacement curve, the modulus of elasticity (MOE) and the modulus of rupture (MOR) were calculated. The number of biological replicates (n) is indicated. Values given are adjusted mean ± standard error of the mean (SEM). Different letters represent significant differences at the 0.05 significance level. For a detailed description of the models fitted to the data and the statistical tests used, see Supplementary Materials and Methods 1.

| **Genotype** | **n** | **Diameter (mm)** | **n** | **MOE (MPa)** | **n** | **MOR (MPa)** |
| --- | --- | --- | --- | --- | --- | --- |
| F_WT_2x | 10 | 30.70 ± 0.98 (A) | 9 | 6918.62 ± 444.24 (A) | 9 | 95.76 ± 3.51 (A) |
| F_WT_4x | 20 | 23.23 ± 0.67 (B) | 20 | 7761.57 ± 287.26 (A) | 20 | 95.38 ± 2.27 (A) |
